# TLR expression in peripheral monocyte subsets of patients with idiopathic inflammatory myopathies: association with clinical and immunological features

**DOI:** 10.1186/s12967-020-02290-3

**Published:** 2020-03-12

**Authors:** Jiram Torres-Ruiz, Daniel Alberto Carrillo-Vazquez, Diana Marcela Padilla-Ortiz, Ricardo Vazquez-Rodriguez, Carlos Nuñez-Alvarez, Guillermo Juarez-Vega, Diana Gomez-Martin

**Affiliations:** 1 Department of Immunology and Rheumatology, Instituto Nacional de Ciencias Médicas y Nutrición Salvador Zubirán, Mexico City, Mexico; 2 Emergency Medicine Department, Instituto Nacional de Ciencias Médicas y Nutrición Salvador Zubirán, Mexico City, Mexico; 3 Universidad de La Sabana, Hospital Militar Central, Bogotá, DC Colombia; 4 Flow Cytometry Unit, Red de Apoyo a la Investigación, Coordinación de Investigación Científica, Universidad Nacional Autónoma de México, Mexico City, Mexico; 5 Tecnologico de Monterrey, Escuela de Medicina y Ciencias de la Salud, Ave Morones Prieto 3000, 64710 Monterrey, Nuevo Leon Mexico

**Keywords:** Dermatomyositis, Monocytes, TLR4, TLR2

## Abstract

**Background:**

Monocytes and toll-like receptors (TLR) have been found in the inflammatory infiltrate of muscle biopsies in patients with idiopathic inflammatory myopathies (IIM), suggesting an important role of these cells in the pathogenesis of myositis. The monocyte subsets, their TLR expression in peripheral blood and their relationship with the clinical characteristics of patients with IIM has not been addressed.

**Methods:**

We recruited 45 patients with IIM diagnosis and 15 age and sex-adjusted healthy controls. We assessed the disease activity and damage, performed a nailfold capillaroscopy and registered the cardio-pulmonary parameters from the medical charts. Monocyte subsets, their expression of TLR2 and TLR4 and the serum Th1/Th2/Th17 cytokines levels were evaluated by flow cytometry. We expressed quantitative variables as medians and interquartile ranges (IQR) or minimum and maximum (min–max). Differences between groups were assessed with Mann–Whitney U and the Kruskal–Wallis tests. Correlation between quantitative variables was assessed with Spearman Rho.

**Results:**

Twenty-nine patients were women (64.4%) and 32 (71.1%) had dermatomyositis. In comparison to healthy controls, patients with active IIM had a higher percentage of intermediate monocytes and lower amounts of classical monocytes. Patients with IIM had a higher expression of TLR4 in all their monocyte subsets, regardless of disease activity and prednisone treatment. Serum IL-6 correlated with the TLR2 expression in every monocyte subset and the expression of TLR2 in intermediate monocytes was higher among patients with dysphagia. Subjects with nailfold capillaroscopy abnormalities had a higher amount of TLR2+ classical and non-classical monocytes and those with interstitial lung disease (ILD) had a higher percentage of TLR4+ non-classical monocytes. The classical and intermediate monocytes from patients with anti Mi2 antibodies had a higher expression of TLR4. The percentage of intermediate monocytes and the expression of TLR4 in all monocyte subsets showed a good diagnostic capacity in patients with IIM.

**Conclusion:**

Patients with IIM have a differential pool of monocyte subsets with an enhanced expression of TLR2 and TLR4, which correlates with disease activity and distinctive clinical features including dysphagia, ILD, vasculopathy, and pro-inflammatory cytokines. These immunological features might be useful as a potential diagnostic tool as well as novel disease activity biomarkers in IIM.

## Background

Idiopathic inflammatory myopathies (IIM) are systemic autoimmune diseases characterized by myositis and extra muscular features [1]. Lymphocytes and monocytes are acknowledged as fundamental effector cells of the pathogenic autoimmune response in IIM [2, 3], since mononuclear cells constitute the main component of the inflammatory infiltrate in muscle biopsies [4]. Monocytes are the circulating precursors of macrophages and dendritic cells and are characterized by their ability to phagocytize, produce cytokines, present antigens [5–7] and their expression of a wide range of toll-like receptors (TLRs), especially TLR2 and TLR4 [8, 9]. In patients with dermatomyositis (DM), polymyositis (PM), immune mediated necrotizing myopathy (IMNM) and anti-synthetase syndrome (AS) macrophages and dendritic cells are prominent in muscle biopsies [10], highlighting the relevance of monocytes in the immunopathology of IIM. Also, the relevance of TLRs in the pathogenesis of inflammatory myopathies has been demonstrated in animal models [11] and muscle biopsies of these patients [12]. In subjects with DM and PM, an enhanced expression of TLR2, TLR4 and TLR9 in the endomysial and perimysial inflammatory infiltrate [13] as well as an overexpression of IFN-γ, IL12p40 and myeloid differentiation factor-88 (MyD88) has been shown in muscle biopsies [14]. Furthermore, the expression of TLR4 correlated with the amount IFN-γ, IL-4, IL-17 and TNF-α in inflammatory cells invading the muscle [13], underscoring the relevance of TLR2 and TLR4 as pro-inflammatory effectors in the pathogenesis of IIM.

In recent years, different monocyte subsets have been acknowledged according to their expression of the LPS receptor (CD14) and the FcγRIII (CD16) and are classified as classical (CD14++/CD16−), intermediate (CD14++/CD16+) and non-classical (CD14+/CD16++) [5, 15]. These monocyte subsets are known to be genetically and functionally distinctive [16], and an expansion of intermediate monocytes has been described in autoinflammatory and autoimmune diseases [15–18]. Notwithstanding the importance of monocytes and TLRs in the pathophysiology of IIM, studies describing the monocytes subsets and their expression of TLR4 and TLR2 in peripheral blood as biomarkers of disease activity are lacking. The aim of this study was to correlate the amount of the distinct monocyte subsets and their expression of TLR2 and TLR4 with the clinical features of patients with IIM.

## Methods

### Clinical evaluation of patients of IIM

We recruited 45 Mexican-mestizo adult patients with DM, clinically amyopathic dermatomyositis (CADM), juvenile dermatomyositis (JDM), AS and PM according to the ACR/EULAR, Bohan and Peter, Connor and Sontheimer criteria [19–22] who were followed-up in a tertiary care center (Instituto Nacional de Ciencias Medicas y Nutricion Salvador Zubiran) from 2016 to 2018 and 15 age and sex-adjusted healthy controls. We excluded patients with any kind of acute or chronic infection, pregnancy, puerperium and neoplasia. All healthy controls and patients signed an informed consent before inclusion and the protocol was approved by our institutional ethics committee (Ref. 2152) in compliance with the Helsinki declaration.

The following disease activity and damage scales were evaluated by a certified Rheumatologist: manual muscle test 8 (MMT8), patient’s and physician’s global disease activity with a visual analogue scale (VAS), the cutaneous dermatomyositis disease area and severity index (CDASI), the myositis disease activity assessment tool (MYOACT and MITAX), and the myositis damage index (MDI) [23]. We registered the type and dose of immunosuppressive therapy. Complete clinical response and remission were defined as the absence of muscular and extra-muscular disease activity for at least six months while taking immunosuppressive therapy or without treatment respectively [24]. Also, we evaluated the presence of dilated, absent or mega capillaries, hemorrhage, thrombosis and neo-vascularization [25] with a qualitative nailfold capillaroscopy, which was performed with a 500× capillaroscope. The presence of interstitial lung disease (ILD) as well as the pulmonary and echocardiographic parameters were registered from the medical charts. We assessed antinuclear antibodies with indirect immunofluorescence in all patients and the myositis specific and associated antibodies with the commercial membrane strip for the detection of antigens EUROLINE (Euroimmune AG, Luebeck, Germany).

### Multiparametric flow cytometry analysis

**Fig. 1:**
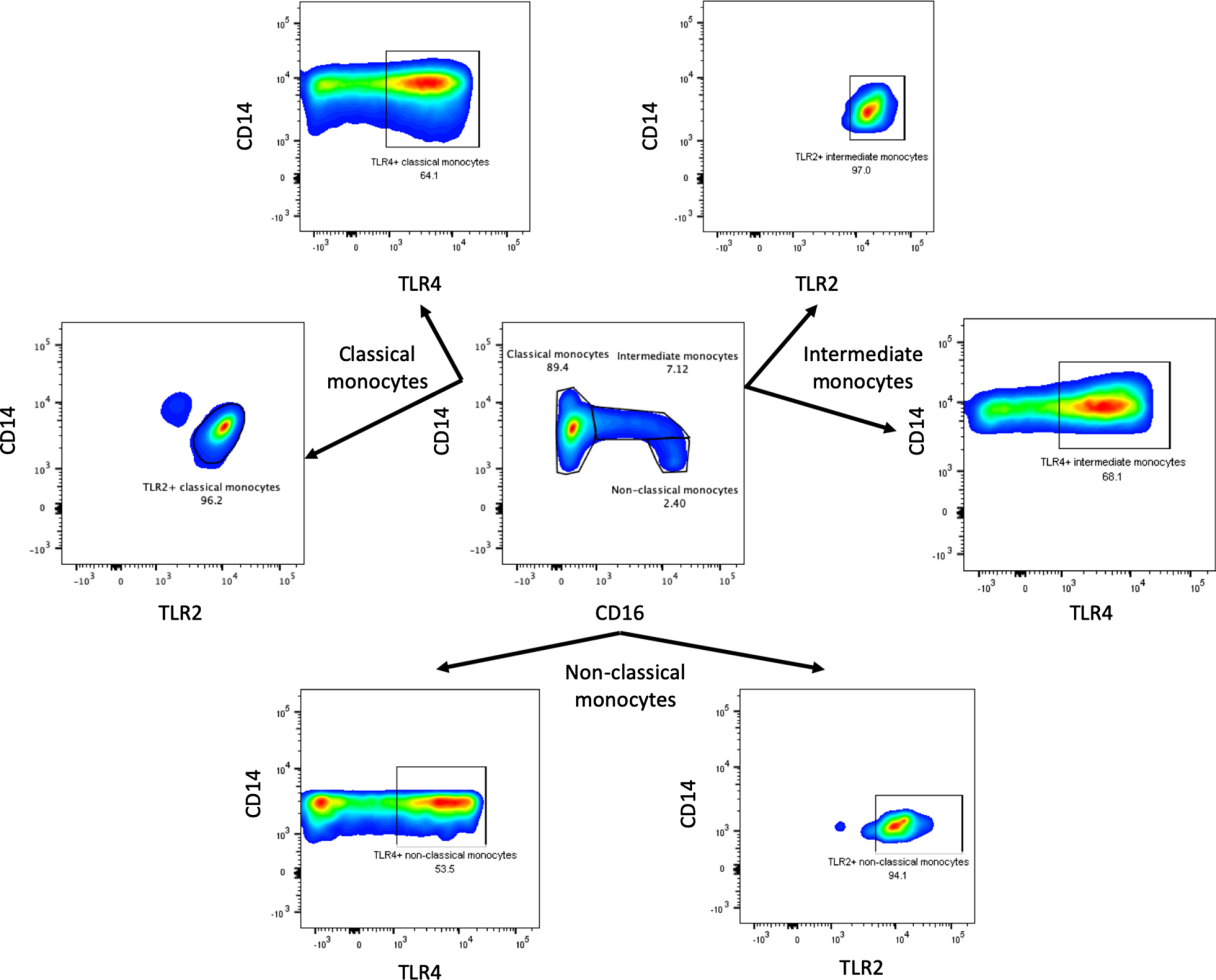
Gating strategy for the monocyte subsets and TLR expression assessment. Classical monocytes were defined as CD14++, CD16−; intermediate monocytes as CD14++, CD16+ and non-classical monocytes as CD14+, CD16++. The expression of TLRs was assessed by measuring the mean fluorescence intensity (MFI) of TLR2 and TLR4 in every monocyte subset

After peripheral blood mononuclear cells (PBMCs) isolation by density gradients with Lymphoprep (Stemcell Technologies, Vancouver, Canada), cells were re-suspended in RPMI with phenol red (Thermo Fisher scientific), washed in PBS with 5% FBS (fetal bovine serum) and stained with the following fluorescent labeled-antibodies: CD14-PerCP (Biolegend, San Diego, CA, USA, catalog number: 325632), CD16-BV605 (Biolegend, San Diego, CA, USA, catalog number: 302040), TLR4-APC (Biolegend, San Diego, CA, USA, catalog number: 312816) and TLR2-BV421 (BD, Biosciences, Franklin Lakes, NJ, USA, catalog number: 565350). The percentage of every monocyte subset was determined according to the International Union of Immunological Societies [26]. The absolute numbers of each subset were calculated taking into account the number of total monocytes from a complete blood count taken at the time of the blood draw. Also, we evaluated the percentage TLR4+ and TLR2+ monocytes in every monocyte subset as well as their expression of TLR expressed as the mean fluorescence intensity (MFI) in arbitrary units (AU). The gating strategy is depicted in Fig. 1. The analysis was performed using the Flow-Jo v10 software. The serum levels of IL-17A, IFN-γ, TNF-α, IL-10, IL-6, IL-4, IL-2 were measured with the cytometric bead array (CBA) human Th1/Th2/Th17 cytokine kit (BD, Biosciences, Franklin Lakes, New Jersey, USA). The data were analyzed with the FCAP array software v3.0 (BD, Biosciences, Franklin Lakes, New Jersey, USA).

### Statistical analysis

We expressed quantitative variables as medians and interquartile ranges (IQR) or minimum and maximum (min–max). Differences between groups were assessed with Mann–Whitney U and the Kruskal–Wallis tests. Correlation between quantitative variables was assessed with Spearman Rho. The analysis was also performed adjusting by prednisone use. To evaluate the usefulness of monocyte subsets and their TLRs expression for the diagnosis of active inflammatory myopathies and to identify patients in complete clinical response we created receiving operating characteristic (ROC) curves, with sensitivity, specificity, area under the curve and 95% confidence intervals (95% CI). A P value < 0.05 was considered as statistically significant. The statistical analysis was performed with the support of the SPSS v25 software (IBM Corp. Armonk, NY, USA).

## Results

### Clinical characteristics of patients with IIM

Twenty-nine patients were women (64.4%). The median (IQR) of age was 46 (37–59) years. Thirty-two patients had DM (71.1%), 3 (6.7%) PM, 4 (8.9%) anti synthetase syndrome and 6 (13.3%) were adults with previous diagnosis of JDM. Regarding immunosuppressive therapy, 29 patients were receiving prednisone (64.4%), 20 (44.4%) methotrexate, 16 (35.6%) azathioprine, 14 (31.1%) hydroxychloroquine, 2 (4.4%) mycophenolate mofetil and 1 (2.2%) cyclophosphamide at the time of blood draw. Immunosuppressants combination was used in 18 patients (40%) and consisted exclusively in the simultaneous use of methotrexate and azathioprine. At the time of evaluation, 30 patients (66.7%) had cutaneous features, 26 (57.8%) had an abnormal nailfold capillaroscopy, 9 (20%) had interstitial lung disease, 7 (15.6%) calcinosis and 5 (11.1%) dysphagia. Nine patients (20%) were in complete clinical response and none of them in remission. The most frequent auto-antibody was anti-Mi2 (22.2%), followed by anti-Ro52 (8.9%). In Table 1, we depict the main disease activity and damage parameters of the patients with IIM.

**Table 1:** Clinical, laboratory and cardio-pulmonary features of patients with idiopathic inflammatory myopathies

Variable	Median (min–max)
Disease activity and damage	
Manual muscle test 8 (MMT8)	144 (45–150)
Visual analogue scale of physician’s disease activity	5 (0–10)
Visual analogue scale of patient’s disease activity	5 (0–10)
Cutaneous dermatomyositis disease area and severity index (CDASI) acute	4 (0–76)
Cutaneous dermatomyositis disease area and severity index (CDASI) chronic	2 (0–21)
Visual analogue scale of constitutional disease activity	0 (0–10)
Visual analogue scale of cutaneous disease activity	0 (0–10)
Visual analogue scale of pulmonary disease activity	0 (0–10)
Visual analogue scale of cardiovascular disease activity	0 (0–10)
Visual analogue scale of other disease activity	0 (0–10)
Visual analogue scale of extramuscular disease activity	3 (0–10)
Visual analogue scale of muscular disease activity	0 (0–10)
Visual analogue scale of global disease activity	5 (0–10)
Total myositis disease activity assessment visual analogue scales (MYOACT)	1.4 (0–7.5)
Total myositis intention to treat activity index (MITAX)	0.85 (0–5.71)
Visual analogue scale of muscular damage	0 (0–10)
Visual analogue scale of skeletal damage	0 (0–9)
Visual analogue scale of cutaneous damage	1 (0–10)
Visual analogue scale of gastrointestinal damage	0 (0–10)
Visual analogue scale of pulmonary damage	0 (0–8)
Visual analogue scale of cardiovascular damage	0 (0–10)
Visual analogue scale of vascular damage	0 (0–5)
Visual analogue scale of endocrine damage	0 (0–10)
Visual analogue scale of ocular damage	0 (0–10)
Visual analogue scale of infection damage	0 (0–10)
Visual analogue scale of malignancy damage	0 (0–5)
Visual analogue scale of other damage	0 (0–10)
Visual analogue scale of global damage	5 (0–10)
Damage extension	0.04 (0–0.52)
Damage severity	0.045 (0–0.5)
Extended damage	0 (0–10)
Health assessment questionnaire (HAQ)	0 (0–3)
Treatment	
Prednisone dose (mg/day)	15 (2–100)
Methotrexate dose (mg/week)	20 (2.5–30)
Azathioprine dose (mg/day)	75 (50–175)
Mycophenolate mofetil dose (g/day)	1.5 (0.5–2.5)
Anti-malarial dose (mg/day)	200 (150–400)
Laboratory and cardio-pulmonary features	
Creatine phosphokinase (U/L)	170 (10–13,325)
Aldolase (U/L)	8.7 (6.2–131)
Alanine aminotransferase (U/L)	31 (5–435)
Aspartate aminotransferase (U/L)	30 (10–1441)
Lactate dehydrogenase (U/L)	226 (45–1243)
C-reactive protein (mg/dL)	0.26 (0.01–13.3)
Erythrocyte sedimentation rate (mm/Hr)	8 (1–56)
Percentage of predicted forced vital capacity	81 (45–108)
Pulmonary artery systolic pressure (mmHg)	32 (20–62)
Percentage of left ventricle ejection fraction	61 (37–76)
Tricuspid annular plane systolic excursion (TAPSE)	20 (11–26)

### Patients with IIM are characterized by a differential profile of circulating monocyte subsets

**Fig. 2:**
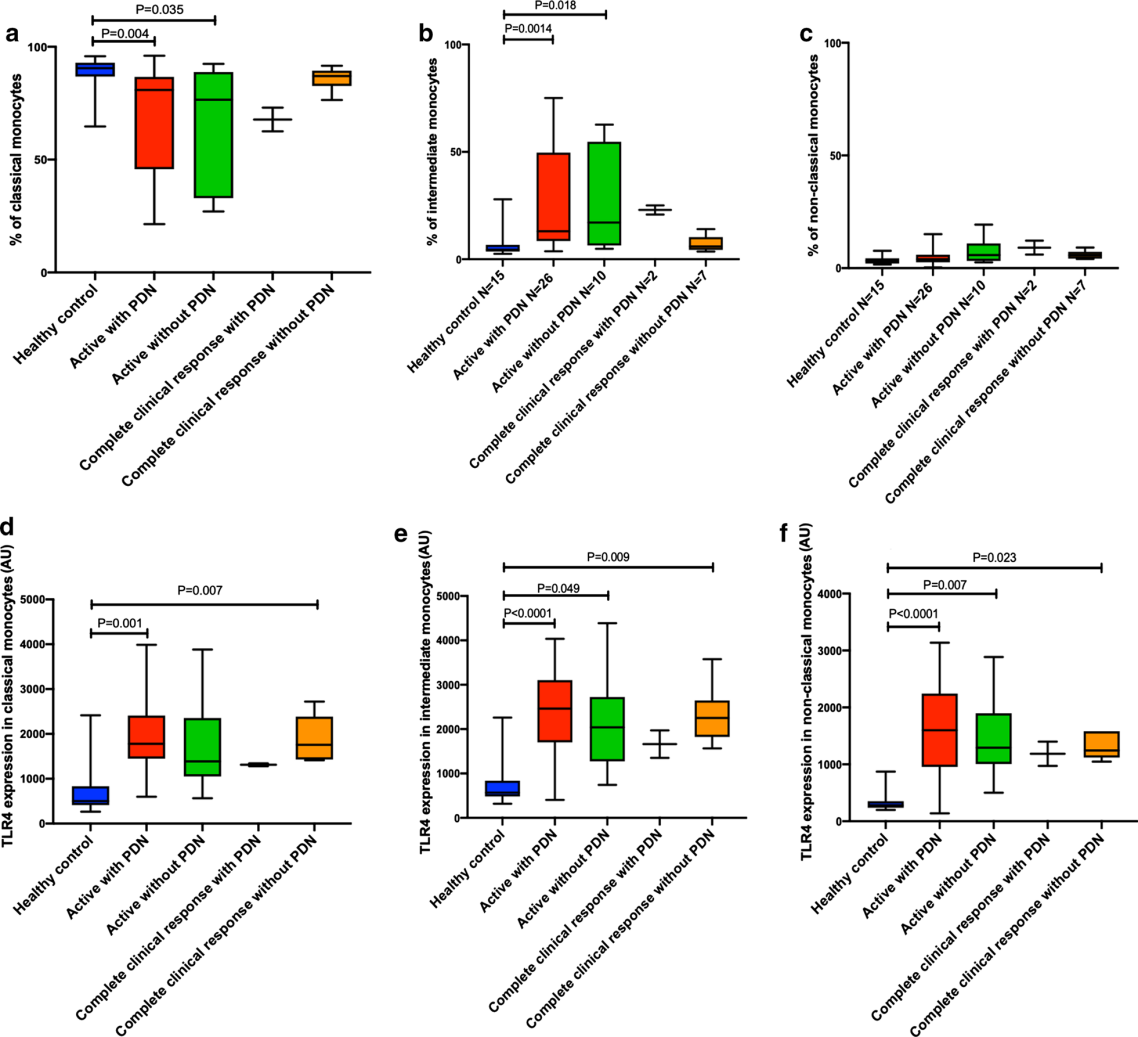
Differential percentage of monocyte subsets and TLR4 expression in patients with inflammatory myopathies according to disease activity. **a**–**c** Patients with IIM and active disease have a lower percentage of classical monocytes (**a**) and higher amounts of intermediate monocytes (**b**). Non-classical monocytes were not different among the study groups (**c**). **d**–**f** All monocyte subsets from patients with IIM have a higher expression of TLR4 regardless of disease activity and prednisone treatment

As shown in Fig. 2, healthy donors had a higher percentage of classical monocytes in comparison to patients with IIM and active disease either using prednisone (90.5% (86.8–92.9%) vs 80.85% (45.78–86.63%), P = 0.004) or without prednisone treatment (90.5% (86.8–92.9%) vs 76.5% (32.9–88.8%), P = 0.035). Likewise, in comparison to healthy donors, IIM patients with active disease and prednisone treatment had a higher percentage of intermediate monocytes (13% (8.45–49.65%) vs 4.49% (3.5–6.71%), P = 0.014) as well as those with active disease without prednisone consumption (17.1% (6.4–54.7%) vs 4.49% (3.5–6.71%), P = 0.018). We did not find a difference in the monocyte subsets nor the TLRs expression in patients receiving azathioprine, methotrexate, cyclophosphamide, mycophenolate mofetil or antimalarials.

The absolute number of classical monocytes inversely correlated with the total myositis disease activity assessment visual analogue scales (MYOACT) (Rho = − 0.429, P = 0.006) and the myositis intention to treat activity index MITAX (Rho = − 0.355, P = 0.027). The percentage of intermediate monocytes was correlated with the VAS of patient’s disease activity (Rho = 0.3, P = 0.05).

### Differential expression of TLR in monocyte subsets among patients with IIM and its relationship with clinical features and circulating cytokines

Classical monocytes from patients with active IIM and prednisone use had a higher expression of TLR4 in comparison to healthy donors (1780 AU (1448–2409 AU) vs 502 AU (412–832 AU), P = 0.001). The same results were obtained when we compared the expression of TLR4 in classical monocytes from patients with IIM in complete clinical response without prednisone treatment and healthy donors (1758 AU (1430–2348 AU) vs 502 AU (412–832 AU), P = 0.007). Expression of TLR4 in intermediate monocytes was higher in patients with active disease and prednisone use in comparison to healthy donors (2464 AU (1701–3105 AU) vs 569 AU (483–837 AU), P < 0.0001) as well as in those with active disease without prednisone treatment (2039 AU (1273–2725 AU) vs 569 AU (483–837 AU), P = 0.049) and in patients with complete clinical response without prednisone treatment (2250 AU (1823–2647 AU) vs 569 AU (483–837 AU), P = 0.009). The expression of TLR4 in non-classical monocytes was higher in patients with active disease and prednisone use in comparison to healthy donors (1598 AU (954–2243 AU) vs 281 AU (237–353 AU), P < 0.0001); as well as in those with active disease without prednisone treatment (1293 AU (1006–1896 AU) vs 281 AU (237–353 AU), P = 0.007) and those with complete clinical response without prednisone use (1243 AU (1118–1580 AU) vs 281 AU (237–353 AU), P = 0.023). Subjects with ILD had a higher percentage of TLR4+ non-classical monocytes (98.25% (97.45–98.93%) vs 95.9% (93.43–98.15%), P = 0.031).

**Fig. 3:**
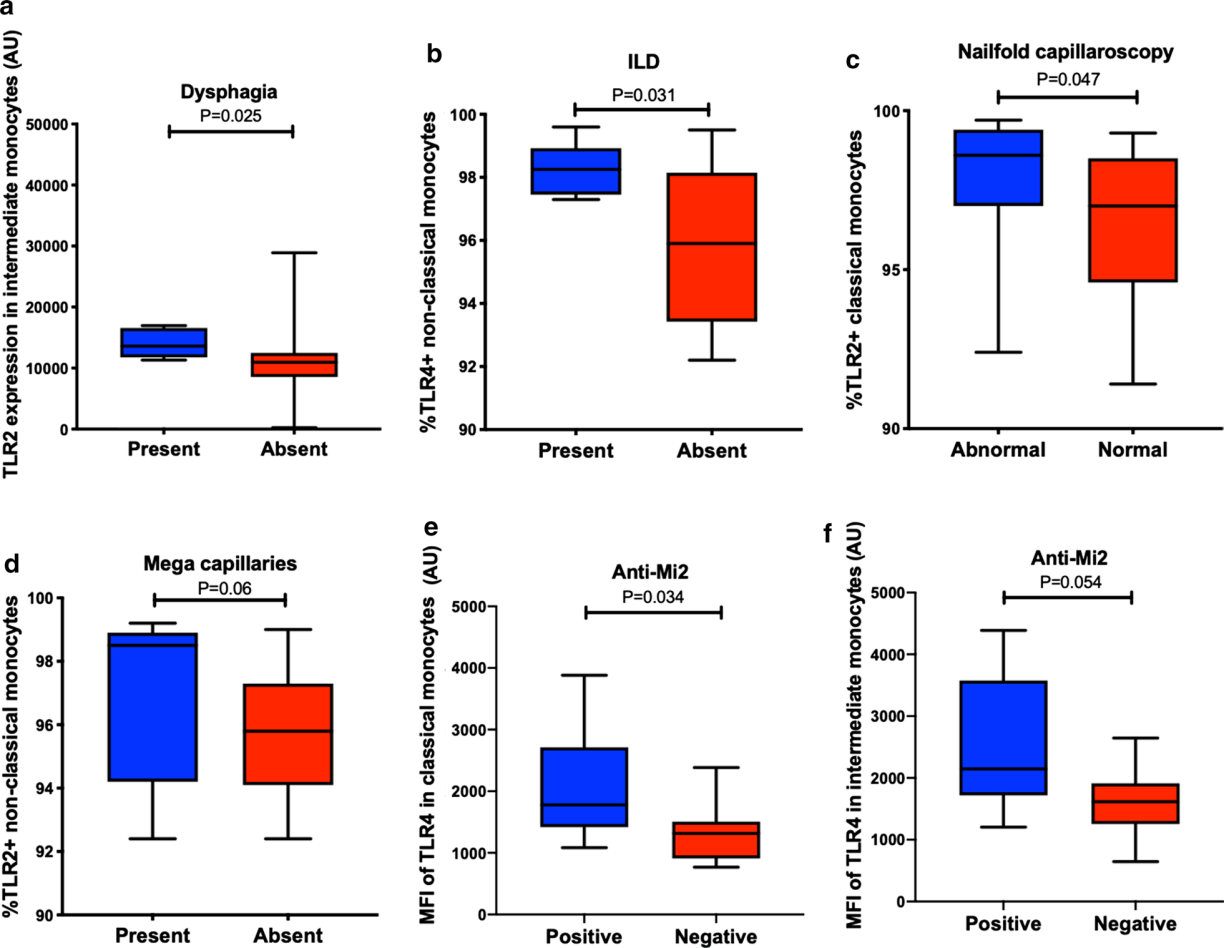
Differential pool of monocyte subsets and TLR expression according to the clinical features. **a** Patients with IIM and dysphagia have a higher expression of TLR2 in intermediate monocytes. **b** Subjects with interstitial lung disease have a higher percentage of TLR4+ non-classical monocytes. **c**, **d** Patients with abnormal nailfold capillaroscopy have a higher percentage of TLR2+ classical monocytes (**d**) and those with mega capillaries a higher percentage of TLR2+ non-classical monocytes. **e**, **f** The expression of TLR4 was higher in classical (**e**) and intermediate (**f**) monocytes of patients with anti-Mi2 antibodies

Interestingly, the expression of TLR4 was higher in classical (1778 AU (1419–2713 AU), P = 0.03) and intermediate (1315 AU (910.8–1506 AU), P = 0.054) monocytes among patients with anti-Mi2 antibodies (Fig. 3). There was a trend towards a significant positive correlation between the percentage of TLR4+ non-classical monocytes and c-reactive protein levels (Rho = 0.457, P = 0.075) and the VAS of pulmonary disease activity (0.52, P = 0.006). The absolute number of TLR4+ non-classical monocytes was correlated with lactate dehydrogenase (LDH) (Rho = 0.7, P = 0.002).

**Fig. 4:**
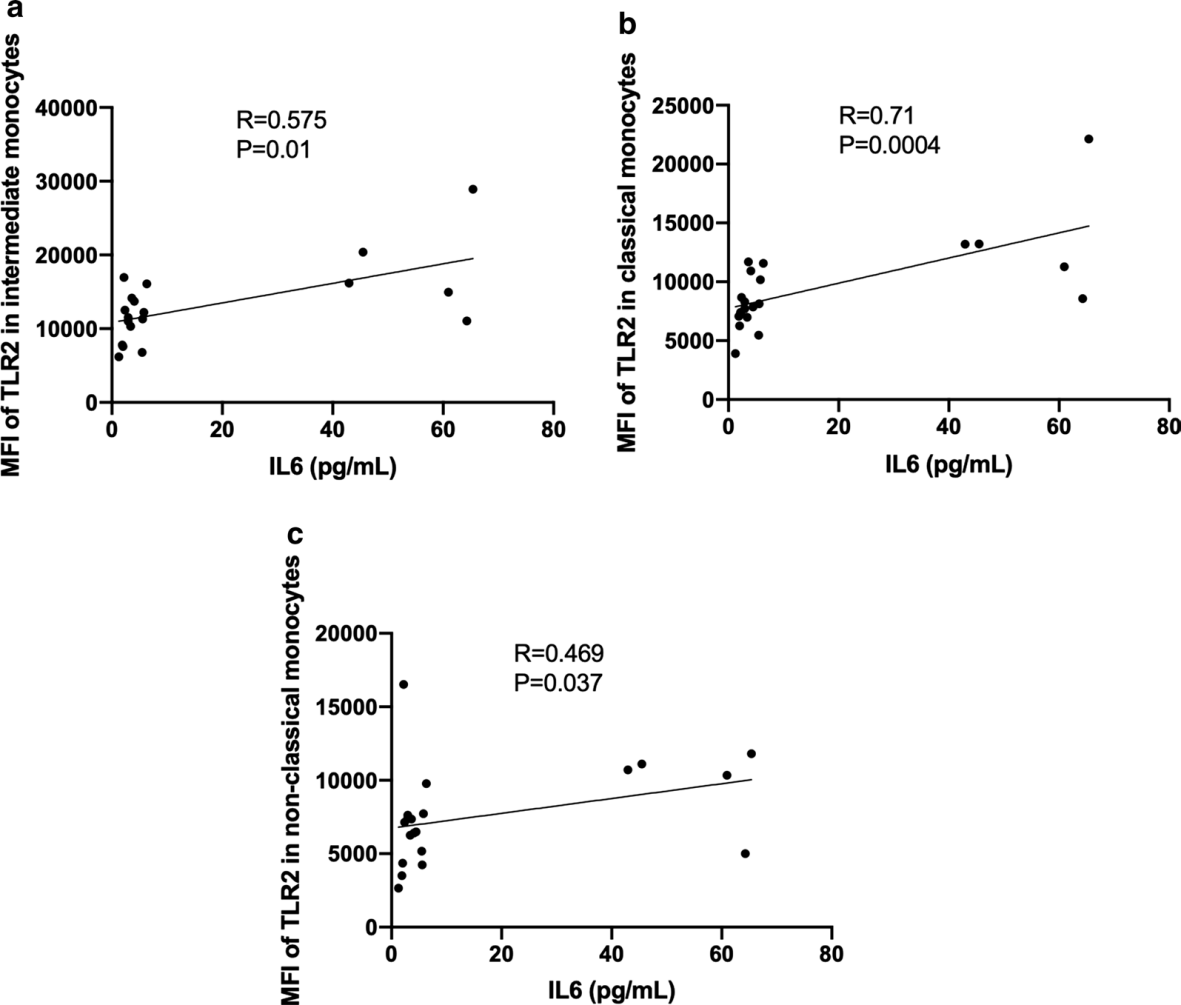
Correlation between TLR expression and IL-6. **a**–**c** The serum levels of IL-6 correlated with the expression of TLR2 in every monocyte subset

Patients with dysphagia had a higher expression of TLR2 in intermediate monocytes (13605 AU (11761–16570 AU) vs 10964 (8543 vs 12487 AU), P = 0.025). The percentage of TLR2+ classical monocytes was correlated with the VAS of gastrointestinal disease activity (Rho = 0.377, P = 0.033. Likewise, patients with abnormal nailfold capillaroscopy had a higher percentage of TLR2+ classical monocytes (98.6% (97–99.4%) vs 97% (94.6–98.5%), P = 0.047) and those with mega-capillaries had a trend towards a higher percentage of TLR2+ non-classical monocytes (98.5% (94.2–98.9%) vs 95.8% (94.1–97.3%), P = 0.06) (Fig. 3). Furthermore, we found that the serum concentration of IL-6 correlated with the absolute number of intermediate monocytes (Rho = 0.395, P = 0.034) and with the expression of TLR2 in all monocyte subsets (Fig. 4). The monocyte subsets and their TLR expression did not correlated with any other serum cytokine. We did not find differences in the monocyte subsets nor the expression of TLRs with respect to the patients’ gender.

### TLR4 expression in all monocyte subsets and the percentage of intermediate monocytes are associated with the diagnosis of inflammatory myopathies

**Fig. 5:**
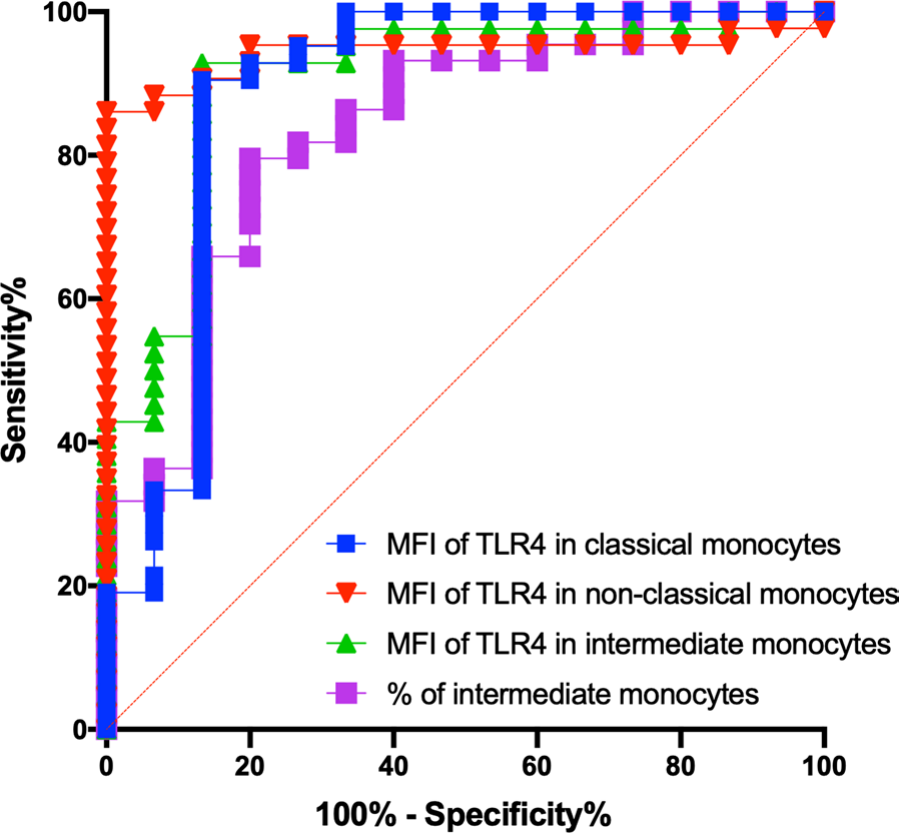
ROC curves for the % of intermediate monocytes and the expression of TLR4 in all monocyte subsets as diagnostic biomarkers in patients with IIM

To test the diagnostic capacity of the monocyte subsets and their TLRs expression, we created ROC curves for each parameter. As shown in Table 2 and Fig. 5, the expression of TLR4 in all monocyte subsets and the percentage of intermediate monocytes are related to the diagnosis of inflammatory myopathies with a high area under the curve, specificity and positive likelihood ratio (LR (+)). Also, a cutoff value of < 4.20% for non-classical monocytes was associated with the diagnosis of complete clinical response with a LR (+) of 4.62, sensitivity (95% CI) of 51.43 (35.57–67.01), specificity (95% CI) of 88.89 (56.50–99.43), and area under the curve (95% CI) of 0.70 (0.55–0.86), P = 0.05.

**Table 2:** Association between monocyte subsets and TLRS expression and the diagnosis of idiopathic inflammatory myopathies

Variable	Cutoff value	Area under the curve (95% CI)	Sensitivity (95% CI)	Specificity (95% CI)	Likelihood ratio (+)	P
Mean fluorescence intensity of TLR4 in non-classical monocytes (arbitrary units)	> 733.5	0.94 (0.88–1)	88.37 (75.52–94.93)	93.33 (70.18–99.66)	13.26	< 0.0001
Mean fluorescence intensity of TLR4 in intermediate monocytes (arbitrary units)	> 2023	0.90 (0.80–1)	54.76 (39.93–68.78)	93.33 (70.18–99.66)	8.21	< 0.0001
Mean fluorescence intensity of TLR4 in classical monocytes (arbitrary units)	> 967	0.88 (0.75–1)	90.48 (77.93–96.23)	86.67 (62.12–97.63)	6.78	< 0.0001
% of intermediate monocytes	> 20.6	0.83 (0.71–0.95)	36.36 (23.78–51.13)	93.3 (70.18–99.66)	5.45	0.0001

## Discussion

The main findings of this study are that patients with IIM have an expansion of circulating intermediate monocytes and that their monocytes subsets have a differential expression of TLR4 and TLR2, which correlate with serum IL-6, as well as with distinctive clinical features. Previously, it was shown that anti-TNF therapy is able to reduce the expression of TLRs in monocytes from patients with rheumatoid arthritis (RA) and spondylarthopathy [27, 28]. Nevertheless, most studies have demonstrated that immunosuppressive therapy does not have an effect in the proportion of monocyte subsets, as shown in patients with RA under methotrexate treatment [29] and in patients with solid organ transplantation [30]. This is in agreement to our results since we did not find a difference in the monocyte subsets nor the TLRs expression according to the immunosuppressive therapy. Nevertheless, prednisone treatment is known to augment the proportion of intermediate monocytes and to diminish the percentage of non-classical monocytes [30], therefore, we decided to adjust our analysis for prednisone intake, confirming that our results are not a consequence of prednisone treatment.

Furthermore, the differential pool of monocyte subsets was maintained in patients with IIM in complete clinical response. Previous studies have shown an increased gene expression of the TLR4 and IFN-γ signaling pathway in patients with inflammatory myopathies [31]. Also, *nuclear factor kappa B (NF*-*κB), tumor necrosis factor a (TNF*-*a), interleukin 1 a (IL*-*1a), interleukin 22 (IL*-*22), toll*-*like receptor 2 (TLR*-*2), toll*-*like receptor 4 (TLR*-*4), toll*-*like receptor 9 (TLR*-*9), interferon alpha (IFNA), interferon gamma (IFNG),* and *retinoic acid inducible gene 1 (RIG*-*1)* are genetic risk factors involved in the pathogenesis of IIM [32]. The constitutional overexpression of pro-inflammatory and TLR-related pathways may explain the differential pool of monocyte subsets and TLRs expression in patients with IIM in complete clinical response. Similar findings have been described in patients with familial Mediterranean fever, in whom increased expression of TLR2 in monocytes has been demonstrated, even during quiescent disease [33].

Similar to our results, lower amounts of CD14++ monocytes have been described in patients with multiple sclerosis (MS) [34] and juvenile idiopathic arthritis (JIA) with enthesitis [35]. Besides, a higher percentage of CD16+ intermediate and non-classical monocytes with a pro-inflammatory phenotype has been described in patients with MS [34], neuromyelitis optica [36], RA [18], SLE [37], ANCA-vasculitis [38], sarcoidosis [39], IgA nephropathy [40], JIA with enthesitis [35], type 1 diabetes mellitus [41], thromboembolism [42], atherosclerosis and stroke [43] which is according to our results. Also, we found that the absolute number of classical monocytes inversely correlated with the disease activity (MYOACT and MITAX), which is according with previous data in patients with RA, where there is a higher percentage of intermediate monocytes during disease activity and a higher proportion of classical monocytes during remission [44]. Our data confirm that a differential proportion of monocytes is found in subjects with autoimmune pathologies, according to disease activity.

Intermediate and non-classical monocytes have been described as proinflammatory [45]. Intermediate monocytes possess phagocytic and pro-inflammatory features, since they secrete IL-1β and TNF-α [37], IL-6 [46] and express higher amounts of TLR 2, 4 and 5 than any other subset. Additionally they express CD80, CD86, HLA-DR and are able to differentiate to M1 macrophages, promote a Th17 response [37] and to induce T-cells proliferation due to their higher expression of CD40 [35]. In contrast, in animal models of muscle injury, non-classical monocytes are recruited in the muscle after tissue damage to promote its repair [47]. Therefore, an expansion of intermediate monocytes may contribute to the pro-inflammatory environment in peripheral blood of patients with IIM, whilst the higher proportion of non-classical monocytes in these patients could be a reflection of muscle damage, since non-classical monocytes are known to respond to CX_3_CL1, which promotes their migration, survival and recruitment in tissues [16].

Regarding TLR expression, a higher expression of TLR2 in monocytes has been described in patients with RA [48], especially in CD16+ monocytes [49] which is according to our results. In IIM patients with nailfold capillaroscopy abnormalities, we found a higher amount of classical and non-classical monocytes expressing TLR2. These data are according to the role of monocytes in endothelial damage in other autoimmune diseases [50]. In the steady state, monocytes patrol the endothelium, but in patients with RA and SLE, it was shown that activated monocytes contribute to vascular damage [50], which could explain the association between this monocyte subset and an abnormal nailfold capillaroscopy in subjects with IIM.

Regarding the relationship between the distinctive monocyte subsets and their TLR expression with the clinical features of patients with IIM, we found a higher percentage of non-classical monocytes in patients with dysphagia and a higher proportion of TLR4+ non-classical monocytes in subjects with ILD. The higher proportion of non-classical monocytes in patients with dysphagia may reflect a more intense and persistent tissue damage, since it is known that this monocyte subset is recruited after muscle injury [47]. According to our results, previous studies have shown that the deficiency of TLR4 decreases pulmonary inflammation and fibrosis in the bleomycin-induced lung injury [51] supporting the relationship between TLR4 and interstitial lung disease found in our study. We found that the expression of TLR2 in all monocyte subsets correlated with serum IL-6. It is known that CD16 + monocytes expressing TLR2 secrete TNF-α, IL-1, IL-6, IL-8, IL-12p40, IL-1Ra and IL-10 after stimuli with lipotheicoic acid [49] confirming the pro-inflammatory profile of these cells in response to pathogen associated molecular patterns (PAMPs).

Regarding the specific and associated myositis antibodies, the monocytes from patients with anti Mi2 antibodies had a higher expression of TLR4+. The importance of TLR4 in DM is highlighted by the presence of TLR4+ cells in the perimysium of these patients [13]. Also, patients with anti-Mi2 antibodies are characterized by intense myositis and an abundant inflammatory infiltrate in muscle biopsy [52]. In this regard, TLR4 is a key mediator of the pathogenic autoimmune and inflammatory response in IIM. In the animal model of myositis induced by intramuscular immunization with histdyl-tRNA synthetase, the TLR4 deficiency suppress the isotype change of the pathogenic autoimmune humoral response in a reaction dependent on Toll/IL-1 receptor (TIR) domain-containing adaptor protein inducing IFN-β (TRIF) [53]. Likewise, in the murine model of myositis induced by immunization with myosin binding protein (C-MBP) and the antibody fusion protein (MYBPC2-MBP), an over-expression of TLR4 and its ligand, the high mobility group box 1 (HMGB1), has been found in muscle biopsies, and it correlated with the expression of major histocompatibility complex I (MHC-I), a key histopathologic finding of inflammatory myopathies [54]. Also, it is known that muscle cells express TLR4 and that their stimulation with HMGB-1 in vitro promotes muscle dysfunction and MHC-I expression [55].

The murine models of myositis have demonstrated the importance of TLR2 and TLR4 in the induction of disease in IIM, since the deficiency of both TLRs [56] or their signaling protein MyD88 completely abolish the disease phenotype [56]. Although these encouraging results suggest a potential therapeutic role of the TLRs inhibition in IIM, it is well known that the TLR2, TLR4 and MyD88 deficiency implies a severe immunodeficiency [57]. In this regard, different peptides have been created to inhibit the TLR4 signaling pathway in animal models of sepsis and mastitis [58, 59] with good results. Inhibition of TLR4 is a therapeutic candidate in autoimmune diseases including IIM. TLR4 has many ligands including heat shock proteins (HSP60, HSP70, gp96, HSP22), HMGB-1, beta-defensin and saturated free fatty acids [60]. Therefore, the inhibition of TLR4 could diminish the inflammatory response secondary to muscle damage in IIM. Nevertheless, inhibition of the TLR4 signaling adaptor molecules such as MyD88, Toll/IL-1 receptor (TIR) domain-containing adaptor protein (TIRAP), TRIF, TIR domain-containing adaptor molecule (TICAM-1) and TRIF-related adaptor molecule (TRAM)/TICAM-2 would result in immunodeficiency since these adaptors are shared by other TLRs [60]. TAK-242 is a TLR4 inhibitor able to suppress the constitutional activation of NF-kB secondary to overexpression of TLR4 [60]. Furthermore, TAK-242 diminish the production of IL-1β, TNF-α and IL-6 and modulates the LPS-mediated secretion of these cytokines in human mononuclear cells without interfering with other TLRs [61] or the TLR4 adaptor molecules [60]. This relatively specific mechanism of action may overcome the infection predisposition as a limitation for the use of TLR4 inhibitors as a therapeutic target in IIM.

Finally, our data suggest that the differential pool of monocyte subsets and their TLRs expression in peripheral blood, may be useful for the diagnosis of IIM and to detect patients in complete clinical response. Previous studies have demonstrated the usefulness of the expression of MHC of class I and II in muscle fibers to differentiate inflammatory myopathies from non-inflammatory myopathies and neurogenic conditions [62]. We found that the expression of TLR4 in all monocyte subsets and the percentage of intermediate monocytes predict the diagnosis of inflammatory myopathies with a high area under the curve and specificity. It would be interesting to explore if these parameters are useful to differentiate inflammatory myopathies from non-inflammatory myopathies or neuropathies. Furthermore, we found a predictive capacity of the percentage of non-classical monocytes for the diagnosis of complete clinical response in patients with IIM. This is a crucial point to avoid excessive treatment in patients in whom persistent muscle weakness is secondary to muscle atrophy instead of disease activity.

Our study has many limitations. First, it is a transversal study with a relatively small sample of Mexican-mestizo patients which may preclude us to find differences regarding the association between disease activity status, the monocyte subsets and the circulating cytokines prospectively. Also, the findings might be limited to the ethnicity of our patients. Nevertheless, it is the first study to address the monocyte subsets in peripheral blood and their relationship with the clinical characteristics and circulating cytokines of patients with IIM.

## Conclusion

All monocyte subsets of patients with IIM have a higher expression of TLR4. The expression of TLR2 in monocytes is related to circulating IL-6 and the presence of dysphagia and ILD is related to a differential expression of TLR2 and TLR4 in CD16+ monocytes. The differential pool of monocyte subsets and their expression of TLR4 are associated with the diagnosis of IIM and the complete clinical response. Further prospective studies are needed to unveil if the circulating monocyte pool is changed after achieving complete clinical response and to unveil the inhibition of TLR4 as a potential therapeutic target in IIM.

## Data Availability

All data generated or analysed during this study are included in this published article.

## Abbreviations

ACR: American College of Rheumatology
ANCA: Anti-neutrophil cytoplasmic antibodies
AS: Anti-synthetase syndrome
CADM: Clinically amyopathic dermatomyositis
CBA: Cytometric bead array
CDASI: Dermatomyositis disease area and severity index
DM: Dermatomyositis
EULAR: European League Against Rheumatism
HRS: Histidyl tRNA synthetase
IFN: Interferon
IIM: Idiopathic inflammatory myopathies
ILD: Interstitial lung disease
IMNM: Immune-mediated necrotizing myopathy
IQR: Interquartile range
JDM: Juvenile dermatomyositis
JIA: Juvenile idiopathic arthritis
LDH: Lactate dehydrogenase
MDI: Myositis damage index
MFI: Mean fluorescence intensity
MITAX: Myositis intention to treat activity index
MMT8: Manual muscle test 8
MYOACT: Myositis Disease Activity Assessment Tool
PBMC: Peripheral blood mononuclear cell
PM: Polymyositis
RA: Rheumatoid arthritis
SLE: Systemic lupus erythematosus
TLR: Toll-like receptors
TNF: Tumor necrosis factor
VAS: Visual analogue scale
